# Supplementary figures and images for: Mechanical evolution of DNA double-strand breaks in the nucleosome
Source: PLoS Comput Biol. 2018 Jun 14;14(6):e1006224. doi: 10.1371/journal.pcbi.1006224 (PMC6025874; doi:10.1371/journal.pcbi.1006224)

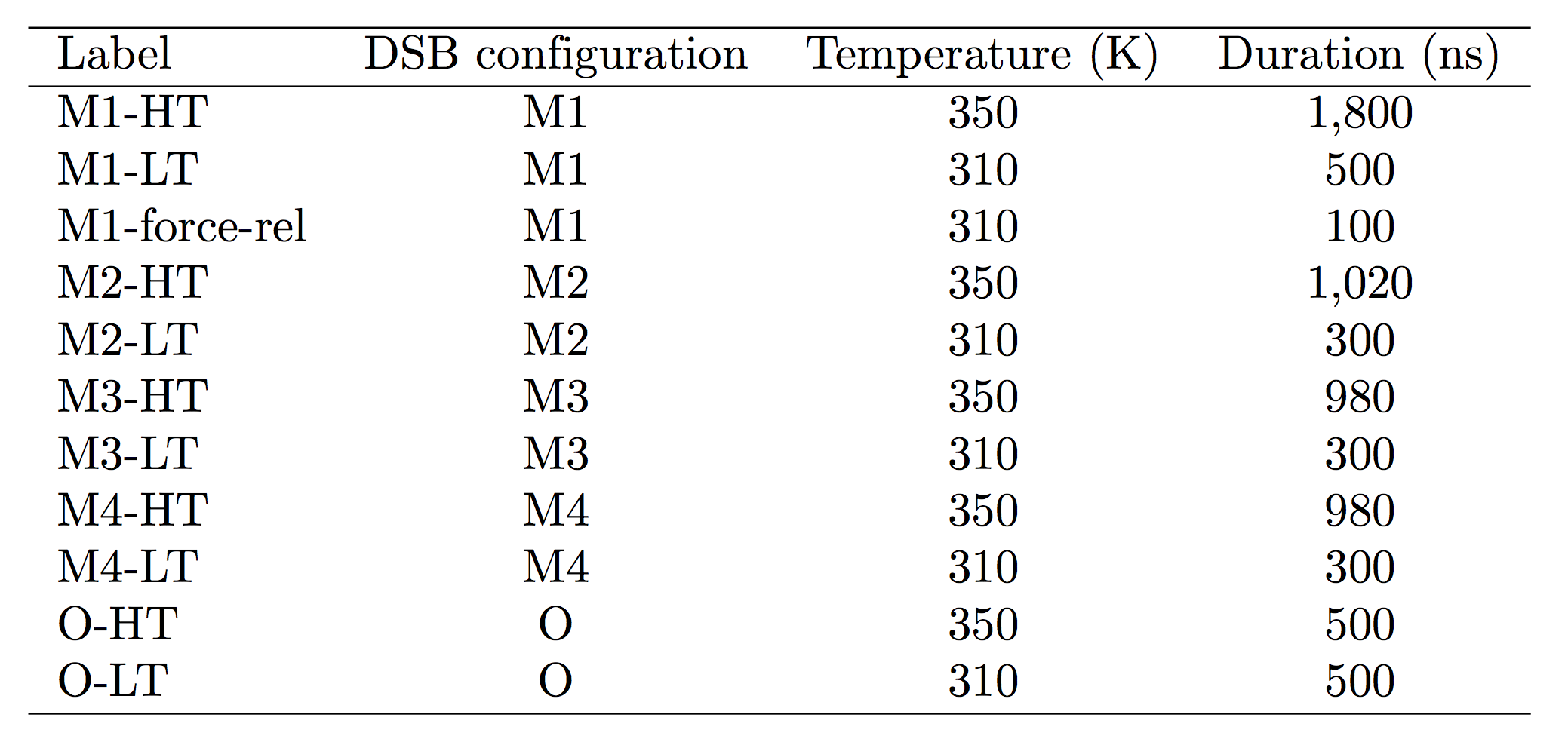

Supplement: S1 Table — All trajectories in this list are simulated with constant-{NVT} (the initial equilibration at constant-{NPT} is omitted). The numerous force-pulling trajectories are not listed. (TIF) [file pcbi.1006224.s001.tif]

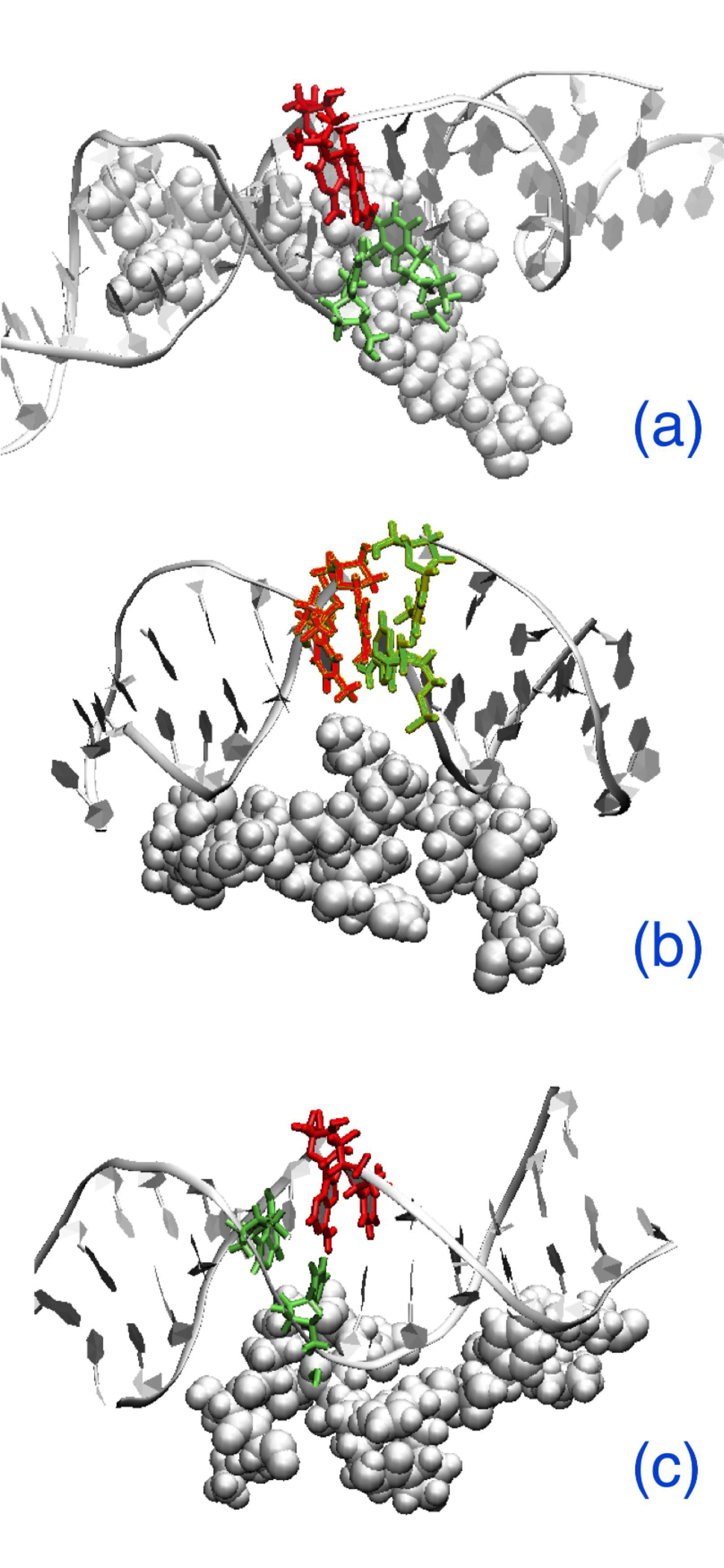

Supplement: S1 Fig — (a) Model M2, with DSB at the outer non-contact site, showing the central A74⋯T114 bp still well bonded. Grey spheres represent a portion of the H3 histone flanking the defect. (b) Model M3, with DSB at the dyad. Grey spheres represent a portion of the H3 tail. (c) Model M4, with DSB at the entry point of nucleosomal DNA. Grey spheres represent a portion of the H3 tail close to the break, which has folded into a double α-helix. (TIF) [file pcbi.1006224.s002.tif]

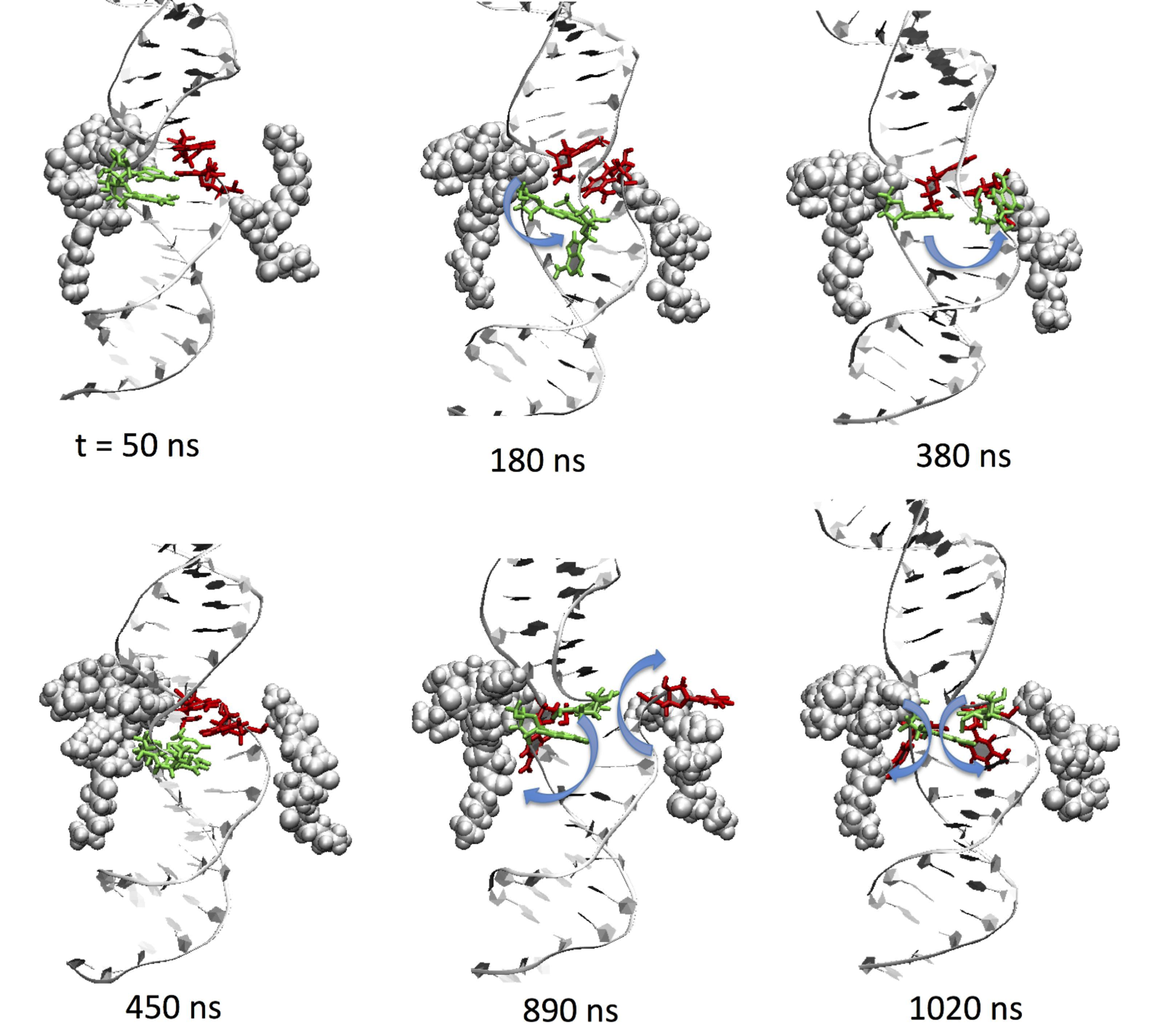

Supplement: S2 Fig — The groups in grey VdW-spheres are the Leu82-Arg83-Phe84-Gln85 of H3, Lys77-Arg78-Lys79-Thr80 of H4 (left side); and Lys9-Gly10-Ser11-Lys12-Lys13, Lys24-Lys25-Arg26 of H2B (right side). DNA bp around the DSB are colored red-green according to the scheme of Fig1b. (TIF) [file pcbi.1006224.s003.tif]

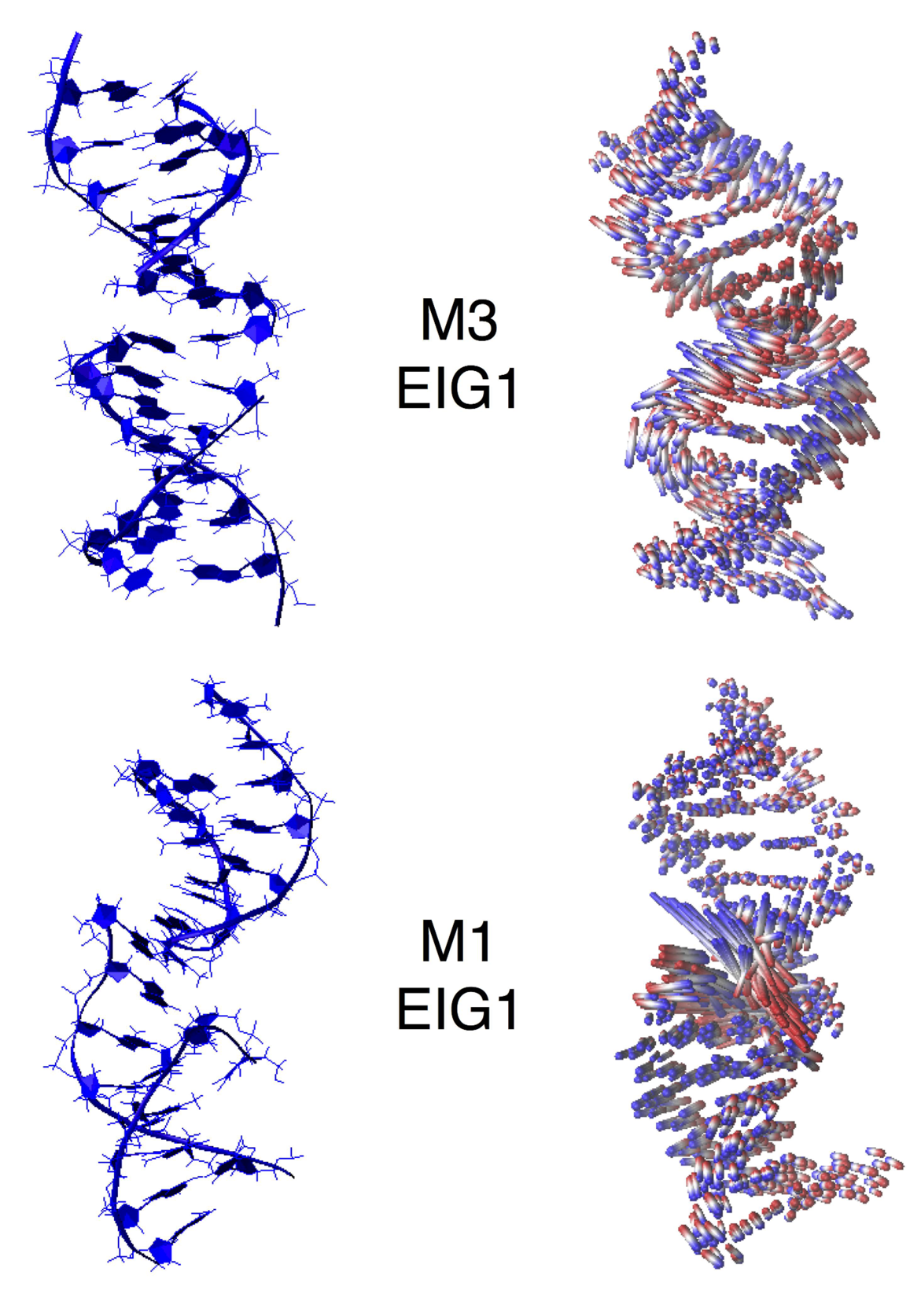

Supplement: S3 Fig — On the left, representative configurations of the DNA fragments close to the DSB, in models M3 (above) and M1 (below). On the right, simultaneous plot of the configurations spanned by the principal motions associated with the first eigenvector, for each model. DNA fragments are aligned with their main axis vertical, the DSB being at the center. The superimposed frames are colored from blue to red, the ordering reflects a virtual motion spanning between the eigenvector extremes. A long stick spanning between the two colors identifies a large motion of the corresponding atom; a shorter stick identifies a local oscillation, of smaller amplitude. (TIF) [file pcbi.1006224.s004.tif]

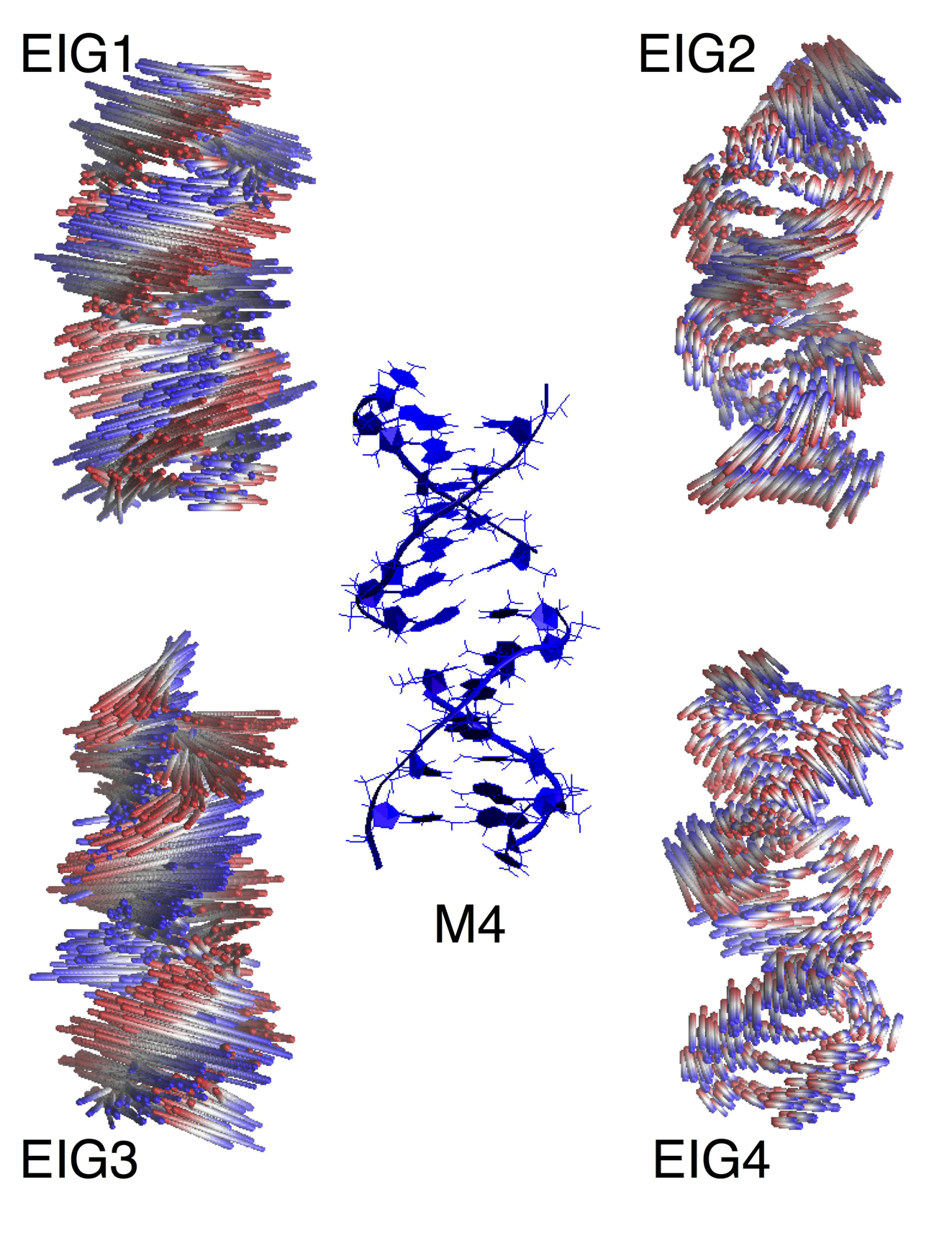

Supplement: S4 Fig — Simultaneous plot of the configurations spanned by the principal motions associated with the eigenvectors 1-4, for the DNA fragment close to the DSB M4 (represented in the central panel). The superimposed frames are colored from blue to red, the ordering reflects a virtual motion spanning between the eigenvector extremes. Also in this case, a long stick spanning between the two colors identifies a large motion of the corresponding atom; a shorter stick identifies a local oscillation, of smaller amplitude. It can be readily appreciated that eigenvectors 1 and 3 correspond to a coordinated, twisting motion of the entire fragment, while eigenvectors 2 and 4 correspond to smaller and less cooperative deformations. (TIF) [file pcbi.1006224.s005.tif]

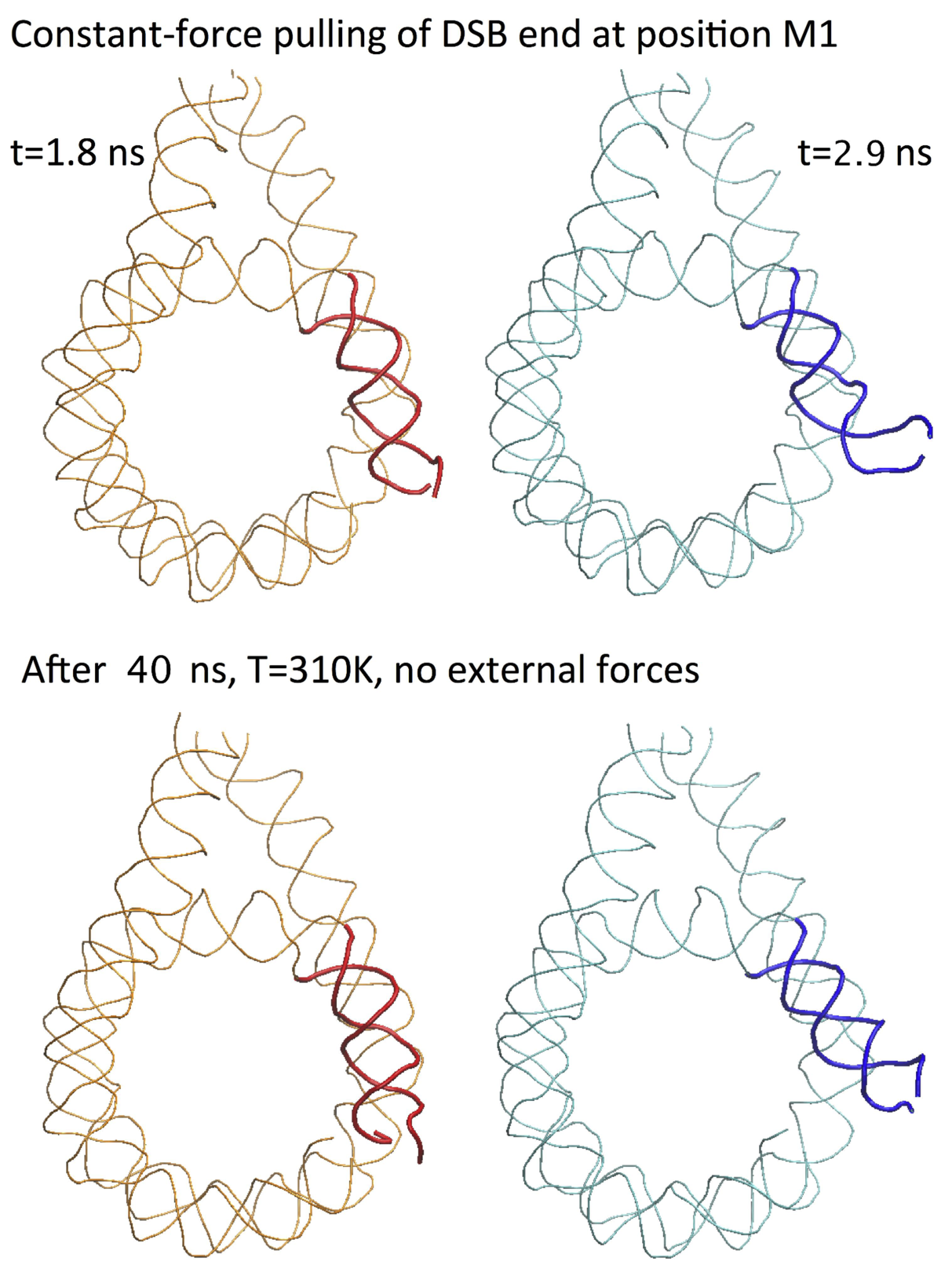

Supplement: S5 Fig — Two configurations extracted from the force-pulling MD simulation of DSB at position M1, at t = 1.8 ns (C180, left, red ribbons) and t = 2.9 ns (C290, right, blue ribbons). The pull force was applied only at the C′-P atoms of the 2 last bp on the upper end of the DSB. The terminal portions of the pulled DSB end are highlighted as a thicker tube, for clarity. Row above: the two configurations at the start of the relaxation. Row below: the two configurations after 40 ns of MD equilibration/relaxation at T = 310 K without any external forces applied. (TIF) [file pcbi.1006224.s006.tif]

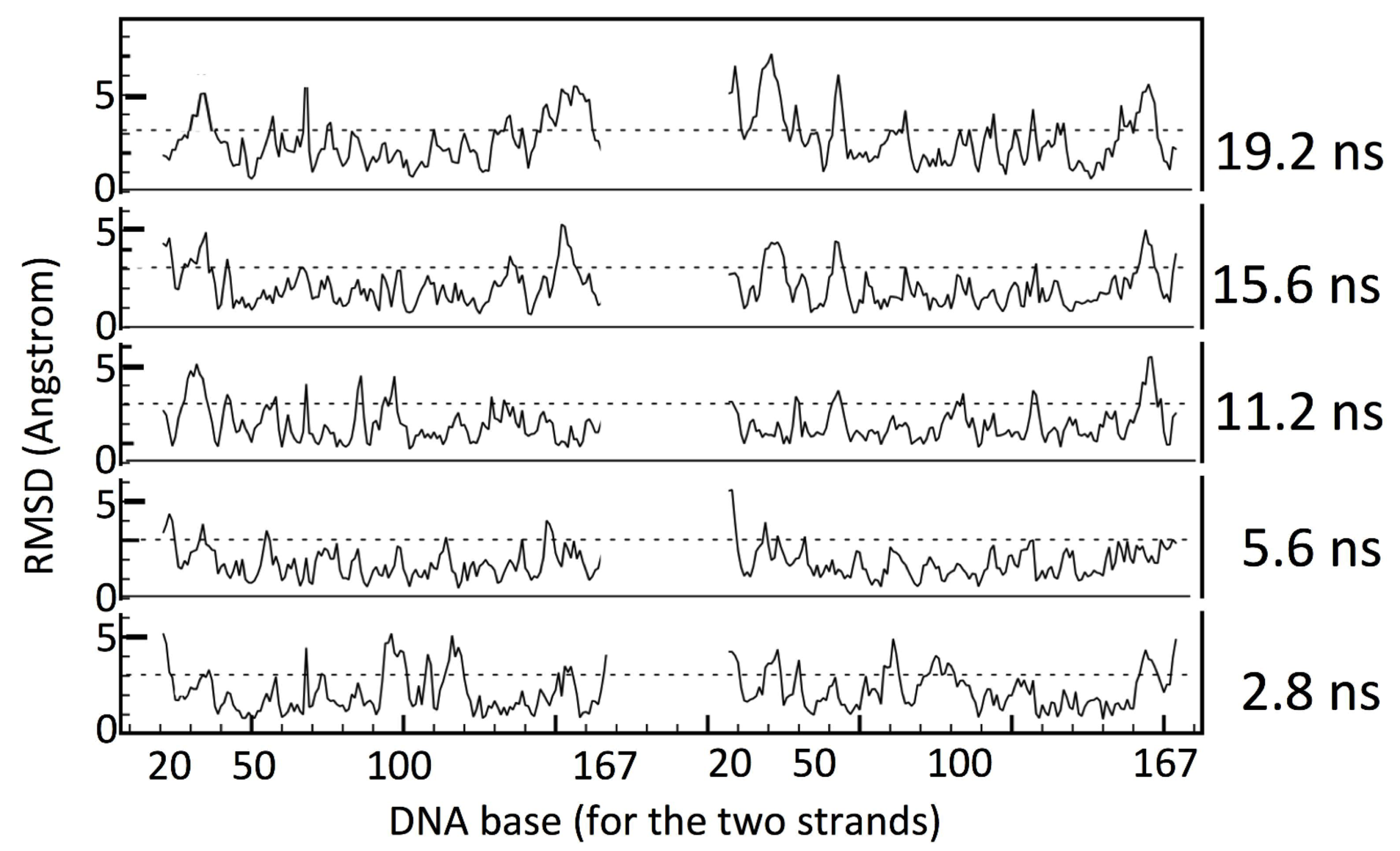

Supplement: S6 Fig — For the M1 trajectory of 1,800 ns, we restarted from the configuration at time 980 ns, and ran 20 ns of trajectory with PME electrostatics; the two parallel segments of trajectory of 20 ns, with cut-off and PME, were then compared, by superposing the structures frame by frame. The plots show the RMSD between the two trajectories for the DNA wrapped in the nucleosome, averaged base-by-base (numbered 20 to 167 for each strand 5′-3′ and 3′-5′), at a few representative times. The dashed lines in each plot indicate the reference value of 3 Å. (TIF) [file pcbi.1006224.s007.tif]

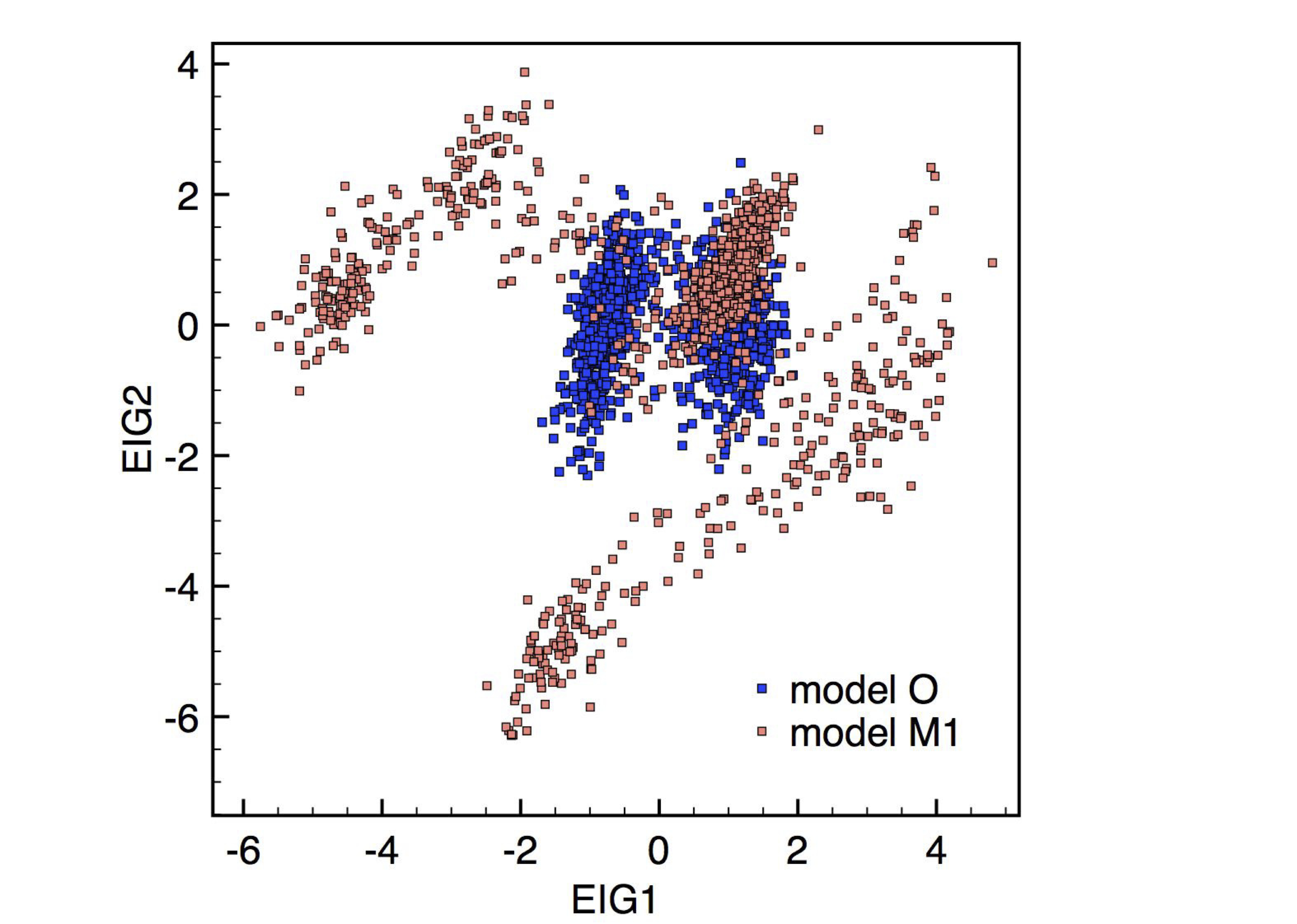

Supplement: S7 Fig — The plot shows the projection of the ∼ 1μs MD trajectory for the DNA fragment comprising bp 62-113 to 75-126, for the pristine nucleosome model O, and for the model M1 including a DSB at this site. Apart from the small region [0:2, 0:2], most likely due to the less mobile base-pairs in the fragment, there is practically no superposition between the essential subspace of the two DNA configurations. This means that the corresponding types of movement are almost entirely different. (TIF) [file pcbi.1006224.s008.tif]

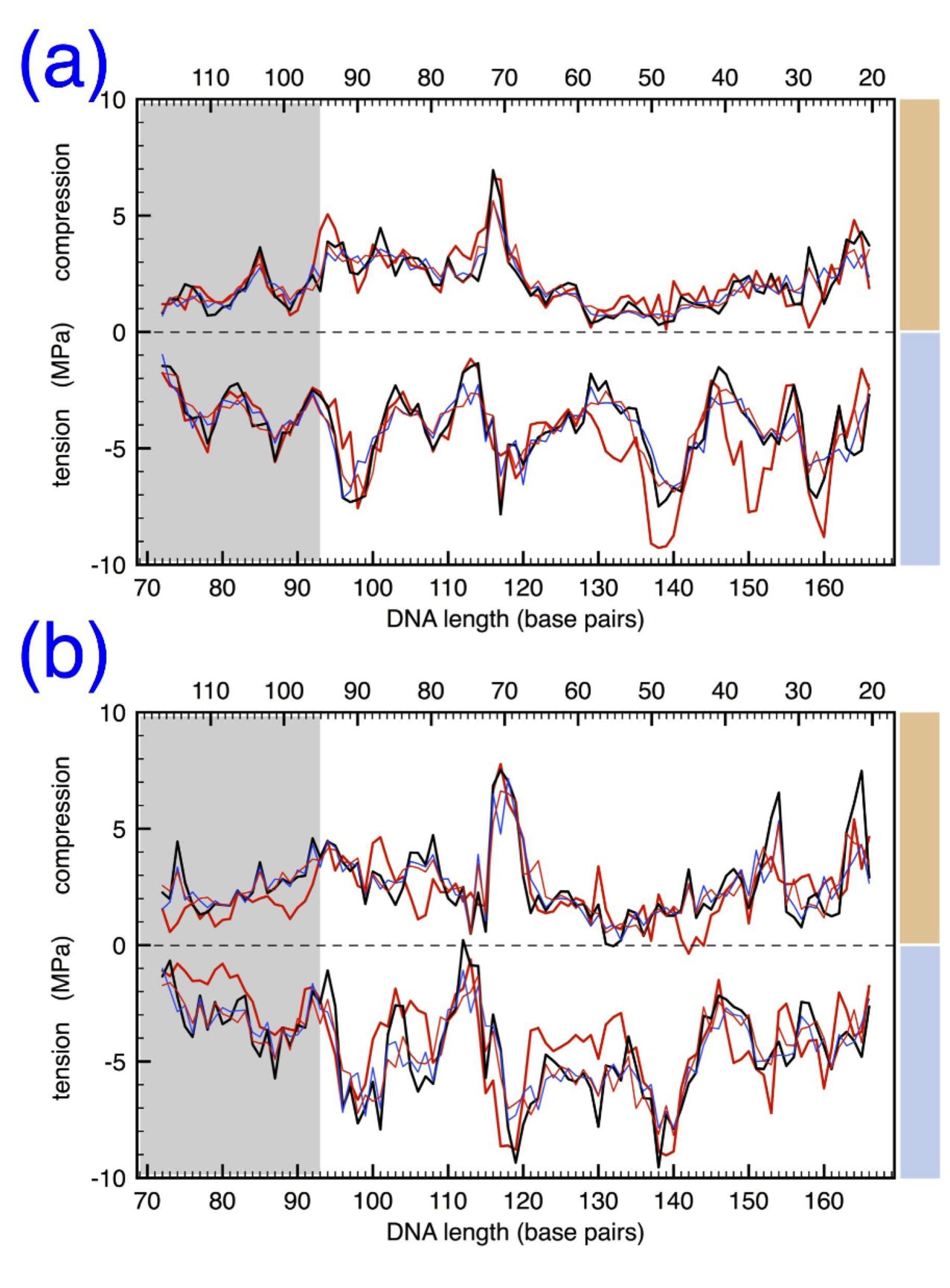

Supplement: S8 Fig — The two plots show the tensile/compressive stress profiles of configurations C185 (a), and C290 (b). With respect to the stress at time t = 0 (thick black lines, same as Fig 8), averaged over 100 frames in the first 1 ns, the thin blue lines show averaging of 200 frames over a double period (0 to 2 ns), and the thin orange lines show averaging the same 200 frames in 8 blocks of 25 frames each. The average RMS deviation is 6-8%. For comparison, the data at t = 40 ns from Fig 8 are also reported, with thick red lines. It is observed that, despite some fluctuation coming from averaging statistics, however when important differences arise between the thick black and red plots (i.e., between t = 0 and t = 40), these are typically larger than the averaging error. See, e.g., bp 70-90 (lower x-axis), or bp 120-135. (TIF) [file pcbi.1006224.s009.tif]
